# Miles with mind: an exploratory study of cognitive-behavioral-based VR training for strategy and motivation in long-distance running

**DOI:** 10.3389/fspor.2025.1722120

**Published:** 2025-11-28

**Authors:** Fernando Pedro Cardenas Hernandez, Jan Schneider, Telse Nagler, Emilia Parada-Cabaleiro, Daniel Schiffner, Andreas Dengel, Hendrik Drachsler

**Affiliations:** 1 Information Center for Education, DIPF - Leibniz Institute for Research and Information in Education, Frankfurt am Main, Germany; 2 Faculty of Computer Science, Goethe University, Frankfurt am Main, Germany; 3 Institute for Psychology, University of Bremen, Bremen, Germany; 4 Department of Music Pedagogy, Nuremberg University of Music, Nuremberg, Germany

**Keywords:** virtual reality, cognitive behavioral approach, mental training, sport psychology, pacing in running, drafting in running, motivation, sports coaching

## Abstract

**Introduction:**

The purpose of this exploratory study was to examine the potential effects of virtual reality (VR) mental training, based on cognitive-behavioral (CB) techniques, on race preparation among long-distance recreational runners within a sports coaching context. Although VR interventions have shown promises for enhancing athletic performance, their integration with CB-based imagery and self-talk remains limited.

**Methods:**

Using a single-subject A-B-A design, six recreational runners completed two races: a first race without mental training and a second race after a series of VR mental training sessions conducted alongside their usual physical training. Each participant used a VR headset equipped with an application that delivered strategy guidance (including pacing and drafting) while also targeting motivation through CB-based imagery and self-talk. Navigation occurred entirely in a virtual environment, with no physical movement. Background audio featured participant-generated self-talk statements. Performance data from VR sessions were recorded through log-file, and emotional responses were assessed with the Emotional Stress Reaction Questionnaire (ESRQ). Race outcomes were compared using smartwatch metrics and participant self-assessments (e.g., Likert scales and open-ended responses).

**Results:**

Following CB-based VR training, most participants reported using pacing and drafting strategies more frequently during the second race. Self-talk frequency increased, and post-race questionnaires indicated higher motivation ratings. Smartwatch data suggested moderately enhanced pacing consistency compared to baseline for some individuals.

**Discussion:**

These exploratory findings suggest that CB-based VR mental training might help the adoption of certain race strategies and encourage self-talk use withing a coaching context. Results from the study may serve as preliminary reference points for future research aimed at integrating VR tools into complementary coaching approaches.

## 1 Introduction

Maximizing athletes' overall development is a crucial objective in many sports, including running, where sports coaching plays a core role. Emerging technologies provide new alternatives to enhance athletes' physical and technical profiles directly influencing their performance and modern coaching methodologies. Wearable devices, such as smartwatches, deliver real-time data on pacing, distance, and heart rate, helping runners and coaches identify areas for improvement and monitor progress in their physical and technical training. Running applications like Strava and Garmin Connect further utilize this data, making it more comprehensive and user-friendly. However, optimizing running performance also requires mental training, a critical focus area in sports coaching for holistic athlete development (1). Virtual Reality (VR) offers a promising avenue for this purpose. VR is a technology that generates computer-simulated environments, creating a compelling perceptual illusion and a sense of immersion that allows users to interact with objects and navigate the virtual space as if they were physically present (2, 3). This capability may make it applicable for implementing mental training by providing controlled scenarios where athletes could develop some essential skills. This approach, known as *VR training*, facilitates embodied simulations that represent and predict actions, concepts, and emotions (4). For runners, VR training could allow simulation of some running conditions, enabling practice in diverse environments aligned with specific coaching objectives, such as preparing for different scenarios and unlimited repetitions (5, 6).

Additionally, VR training could be valuable for training programs targeting skills that may be costly, impractical, or risky to practice in real-life settings, including sport (7). Besides environmental simulations, VR may also provide preliminary utility for training certain cognitive and mental skills, potentially offering additional dimensions for different sports (8).

The development of mental skills through VR training interventions typically considers both cognitive and behavioral elements (9). Cognitive behavior therapy is commonly defined as a structured, time-limited, and present-focused psychotherapeutic approach that seeks to address current difficulties by identifying and modifying maladaptive thoughts and behaviors (10). More broadly within clinical psychology, cognitive behavior therapy refers to a diverse coherent set of therapeutic techniques designed to promote changes in cognition and behavior (11). Beyond these two aspects, cognitive behavior therapy also considers affective and motivational responses influenced by external stimuli, such as emotionally charged words (12). A similar integrative approach is applied in sport psychology, where cognitive behavioral (CB) techniques serve as structured interventions within coaching practices. These techniques help athletes transform dysfunctional performance behaviors into functional ones through systematic training (13). Among CB techniques, two stand out for their relevance, frequent application in sport performance, and importance in coaching strategies: *imagery* and *self-talk*. Imagery involves the ability to adjust, generate or replicate mental scenarios from memory to achieve cognitive, emotional or behavioral effects (14). Self-talk refers to verbalized or internal dialogue that guide, motivate, and regulate an athlete's responses to events (15).

Previous research has indicated potential associations between VR training and outcomes such as strategic thinking, decision-making, adaptation to unexpected events, and psychological resilience in sport (16). In addition, findings from exercise settings have suggested that VR may also affect motivational responses, which play a role in maintaining training engagement (17). However, the integration of VR with CB-based techniques has received limited attention in the sport domain. This approach may offer opportunities to support both intrinsic motivation, conceptualized as engagement driven by inherent enjoyment and personal satisfaction (18), an important factor associated with athletic performance (19, 20), and the mental preparation of race strategy. Race strategy, which involves pre-planned elements for competitive preparation (21), comprises two key components: *pacing*, the regulation of running speed to optimize energy expenditure throughout a competition, leading to identifiable pacing profiles (22), and *drafting*, the tactical positioning behind other runners to minimize air resistance, with effectiveness dependent on spacing and formation among runners (23).

Despite sport coaches emphasizing the importance of motivation and race strategy preparation for athlete development and competitive success, existing training tools remain limited (1). Traditional mental training approaches (such as visualization journals, guided meditation, and in-person coaching sessions) offer some benefits but often lack immersion, engagement, and real-time feedback, constraining their effectiveness. VR-based tools emerge as a possible solution to address these gaps, enhancing scalability and real-time adjustments in coaching contexts. VR experiences can also provoke varying intensities and durations of emotional responses (both positive and negative emotions), a phenomenon known as emotional reactivity. Some evidence suggests that certain VR scenarios may trigger stress responses, defined as psychological or physical stimuli that disrupt homeostasis, which could potentially attenuate the intended benefits of such interventions (24, 25). This highlights the importance of evaluating emotional responses during the implementation of VR-based training.

To support the development of CB training tools in sports coaching and evaluate their impact on athletic performance, we created a CB-based VR application for runners. This application integrated imagery and self-talk to help athletes mentally prepare for races and strengthen their motivational mindset. The CB-based VR application did not require physical running or walking for virtual navigation. It used imagery through: (1) its cognitive function, enabling mental rehearsal of pacing and drafting to prepare for actual races, and (2) its motivational function, regulating arousal by familiarizing runners with race scenarios to manage emotional states (26). Similarly, self-talk was implemented through: (1) its instructional dimension, providing technical guidance with race strategy statements, and (2) its motivational dimension, fostering motivation through encouragement cues (15).

We conducted a study using a *single-subject A-B-A design* with six participants to explore the effects of CB-based VR training, delivered over multiple sessions with our VR-based application, on athletic performance. This design is an established approach in sports psychology for evaluating conventional CB techniques, with prior studies demonstrating its utility across diverse sports such as swimming (27), basketball (28, 29), and archery (30).

This empirical foundation supports its suitability for evaluating how a CB-based VR training may influence both performance and the psychological factors that support performance in runners.

Accordingly, our psychological assessment focused on three areas: (1) *emotional reactivity*, measuring potential stress responses to VR training; (2) *changes in core mental toughness attributes*, concentration (sustained focus during challenges), confidence (beliefs in one's abilities), and commitment (resilience to adversity) (31), to explore VR training potential contributions in these attributes; and (3) *subjective race perceptions* of the motivation, physical state and performance achievement, offering deeper insight into participants’ experiences.

This study was guided by these research questions (RQ's):

- **RQ1**: How do participants execute the instructed race strategy pacing and drafting during their VR training with the CB-based VR application?
- **RQ2**: How does the CB-based VR application influence participants' self-reported emotional reactivity during their VR training?
- **RQ3**: How do VR training sessions affect participants' self-assessments of CB skills (imagery and self-talk), mental toughness attributes (concentration, confidence, and commitment), and subjective race perceptions (motivation, physical state, and performance achievement) in the next competition?
- **RQ4**: How does the VR training influence participants' self-reported use of race strategy in the next competition?

## 2 The CB-based VR application

### 2.1 Foundation

The application of VR for mental training in sport, while innovative, builds upon a substantial foundation in clinical research. It is crucial to situate this work within the robust and well-established body of evidence demonstrating VR's efficacy in delivering psychological therapies, particularly cognitive behavioral therapy, for clinical populations. Decades of work with *virtual reality exposure therapy* has shown meaningful improvements in conditions such as anxiety disorders, phobias, and post-traumatic stress disorder by enabling individuals to confront challenging stimuli in a controlled and safe environment (32–36).

The novelty of the present research, therefore, lies not in the use of VR for psychological intervention in itself, but in its deliberate adaptation from a clinical context to a sport performance-enhancement domain.

While clinical VR primarily focuses on alleviating distress and supporting the restoration of functional levels, its use in sport psychology seeks to extend athletes' capabilities by targeting psychological elements (57, 58). This represents a significant shift in objectives, from remediation in clinical context to performance development in sport.

Furthermore, VR may offer transformative advantages over conventional sport psychology methods, including dynamic simulations of competitive environments and the potential for scalable, standardized interventions that overcome typical logistical and accessibility constraints. However, potential issues such as cybersickness, which is a visually induced form of motion sickness that occurs during exposure to virtual environments and is characterized by symptoms such as nausea, disorientation, and dizziness (37), must be mitigated. There is also a risk of oversimplifying the multifaceted, relational practice of sport psychology into a technological fast solution, which would fail to capture the field's inherent complexity.

### 2.2 Implementation

The VR application used in this study builds upon a previous user experience study (38). Based on those findings, we addressed issues such as flickering and symptoms of cybersickness. We also tested arm swinging as alternative navigation method but ultimately retained thumbstick-based movement as the most suitable solution for this research context.

The CB-based VR application was developed in Unity for the Meta Quest 2 VR headset and delivered entirely in English. We used the CityGen3D editor for Unity to generate city streets for some VR scenes, created the avatar's voice using a text-to-speech voice generator with an “excited” voice style, and applied Unity's built-in rendering pipeline to develop the VR application. The application was installed on the Meta Quest 2, featuring a resolution of 1,832 × 1,920 pixels per eye, a 90 Hz refresh rate, and a 90-degree field of view. Audio was delivered through the Meta Quest 2's integrated headphones. To use the application, participants needed to stand up within a virtually delimited boundary. Navigation in the virtual environment was controlled by pushing the left thumbstick in the desired direction (no physical running or walking was required). Rotation was achieved by turning either their head or entire body, depending on their preference.

#### 2.2.1 The VR environment

The VR environment featured *three* distinct types of scenes: setting, coaching, and competition.

**Figure 1:**
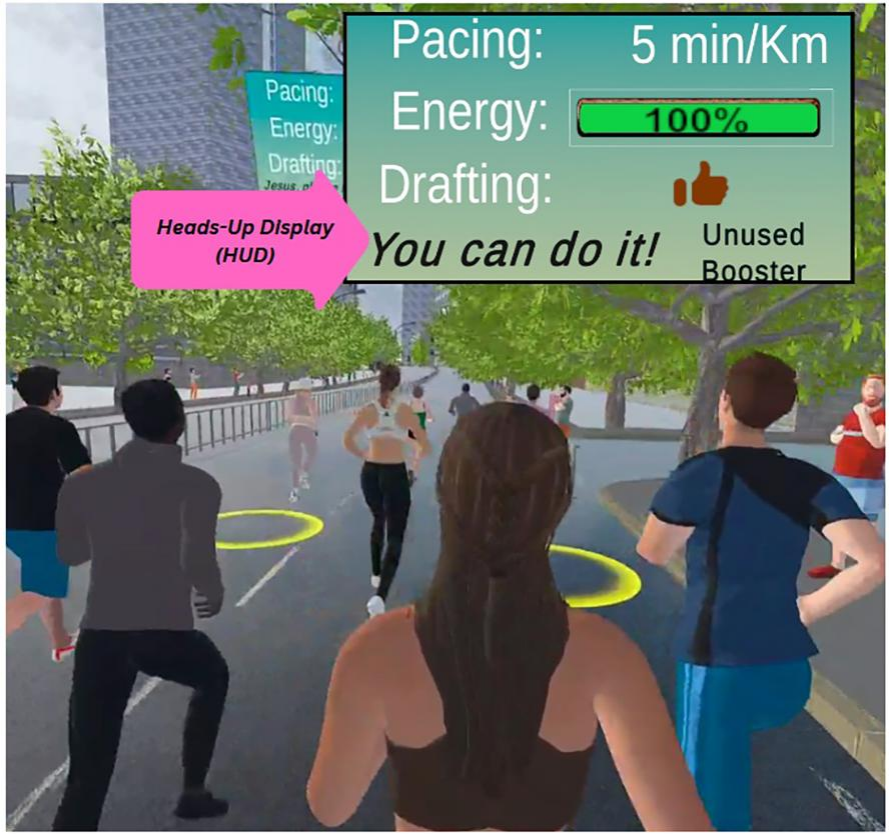
Competition scene 2 displaying drafting spots in yellow circles. The HUD is shown in an enlarged view in the top-right corner. It provides information on participants’ pacing, energy, drafting status, booster status, and stage-specific self-talk.

*Setting scene*: This unique scene allowed participants to establish their initial running speed, reflecting the pacing they aimed to maintain during a race, which then served as a reference for their VR training.
*Coaching scenes*: There were five coaching scenes, each featuring a virtual coach that provided encouragement, guidance and essential instructions for the upcoming competition scenes. Afterward, participants proceeded to the next scene.
*Competition scenes* (See Figure 1): There were four competition scenes, each representing common stages of a race: *start, first half, second half,* and *end*. Each scene features virtual non-playable runners. However, their number gradually decreased to simulate the natural reduction in visible competitors commonly observed as a race progresses. During these scenes, participants could adjust their pacing by using a button in the right controller. The virtual coach provided voice guidance but was not visually present. Each competition scene incorporated background music and personalized self-talk (See Educational Phase Section). The background music played continuously, while the self-talk repeated after the coach delivered an instructional and motivational speech. Upon reaching a specific position within each scene, participants were automatically transitioned to the subsequent coaching scene.

A Heads-Up Display (HUD) (see Figure 1) was integrated into all competition scenes, serving as a central interface that provided participants with immediate performance indicators and status updates. The HUD showed the *pacing level*, which indicated participants' pacing within the virtual environment, and the *energy level* which reflected their remaining energy reserves. It also included the *booster status*, which, when activated, elevated participants' pacing to a maximum of 2:30 min/km for a unique duration of 30 s. After activation, pacing sharply declined with no opportunity for recovery during the ongoing session. The *drafting formation status* (only in competition scenes 2 and 3) represented the effectiveness of participants' drafting formation. The *self-talk feature* offered individual affirmations to remember or internalize during specific scenarios and was available in all competition scenes.

**Figure 2:**
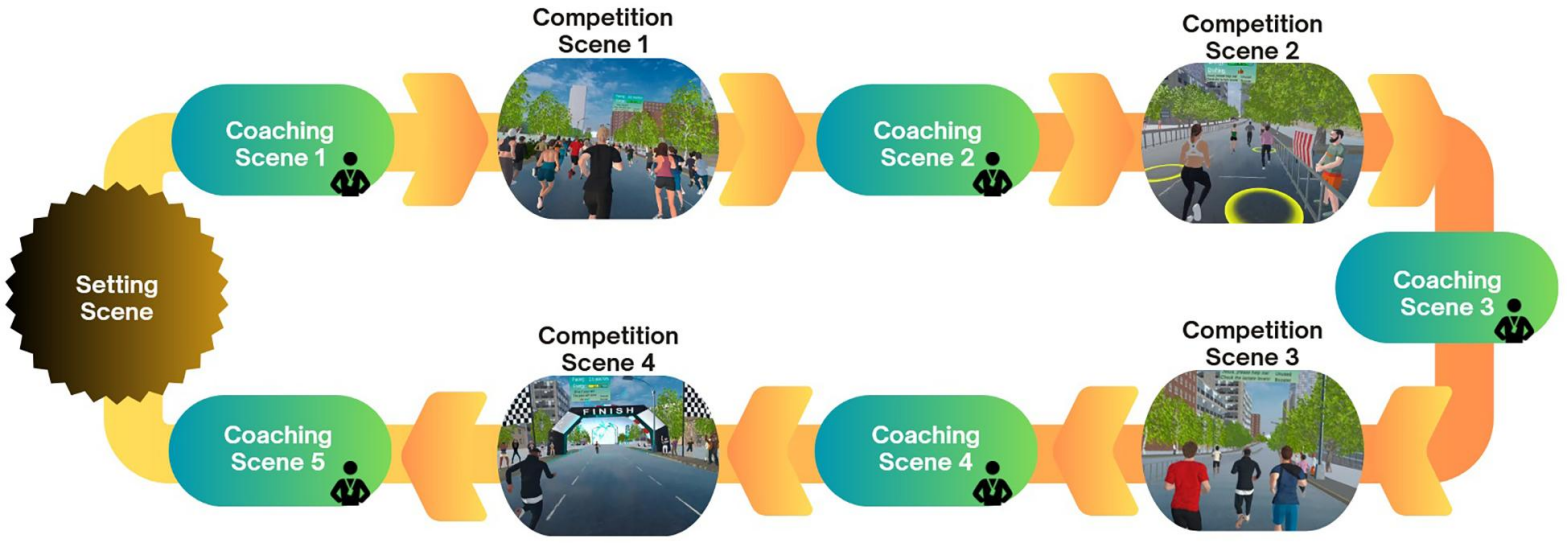
Sequence of scenes in the CB-based VR application.

Figure 2 illustrates the sequential arrangement of virtual scenes in the CB-based VR application. The VR application maintained fixed scene features throughout the study, ensuring all participants experienced identical and repetitive training scenarios without performance-based adaptations. Detailed descriptions of each scene are provided below.

##### 2.2.1.1 Coaching scene 1

In this scene, the virtual coach emphasized the dual importance of mental and physical training in race preparation. The coach also mentioned that visual and auditory cues would be provided throughout the scenarios to sustain motivation and signal the implementation of a race strategy. Additionally, it encouraged staying relaxed and focused on the start of the race, using a conservative pacing to manage energy efficiently, and employing self-talk to maintain motivation. Participants had to cross a door to proceed to the next scene.

##### 2.2.1.2 Competition scene 1

This scene simulated the *start of the race*, where participants competed against *52* virtual runners, all awaiting the countdown gunshot to begin. The virtual coach emphasized that the real challenge is finishing the race with resilience, not just starting it fast. Moreover, it advised participants to focus on running their own race, adjusting and maintaining a steady pacing, aiming for their personal average pacing or slightly slower.

##### 2.2.1.3 Coaching scene 2

In this scene, the virtual coach explained drafting as a strategy to reduce wind resistance by positioning oneself behind other runners. The coach introduced two common drafting formations: *Formation-1* involves standing behind another runner at approximately 1.2 meters, and *Formation-2* involves positioning oneself equidistantly between two runners, each 1.2 meters away. Participants were instructed to try these formations in the next competition scene. They would receive one thumb-up for successfully executing Formation-1 and two thumb-ups for Formation-2, reflecting the effectiveness of each formation. To help participants achieve the correct position for Formation-2, the virtual coach mentioned that glowing circles between two virtual runners would indicate where they should position themselves. A whiteboard on the left side of the coach summarized the formations and their score system. Finally, participants were encouraged to rely on their positive self-talk and interrupt potential negative thoughts. This was simulated by shattering virtual glass panels displaying these thoughts. Breaking the panels not only cleared their path in the virtual environment but also symbolized overcoming mental barriers through the VR experience.

##### 2.2.1.4 Competition scene 2

This scene simulated the *first half of the race*, where participants competed against *20* virtual runners. The virtual coach encouraged them to identify opportunities to overtake slow runners and to do so steadily and confidently using drafting. In this scene, the glowing circles for Formation-2 were visible (see Figure 1). The coach also emphasized the importance of saving energy for the final push by employing both drafting and pacing.

##### 2.2.1.5 Coaching scene 3

In this scene the virtual coach announced that the next scene would be the second half of the race, focusing on drafting skills once again. This time, participants would rely on their own judgment without markers or hints. The coach expressed strong confidence in the participants' ability to handle this challenge. Motivational quotes were displayed within the virtual environment to inspire participants and boost their motivation.

##### 2.2.1.6 Competition scene 3

This scene simulated the *second half of the race*, where participants competed against *eight* virtual runners. The virtual coach emphasized that determination and commitment are especially crucial at this stage due to the inevitable fatigue. Besides, it advised participants to stay motivated and maintain effective pacing and drafting.

##### 2.2.1.7 Coaching scene 4

In this scene, the virtual coach informed participants that the final part of the race was next, encouraging them to take on this ultimate test and prove their readiness. To go to the final scenario, participants had to walk up a spiral ramp, punching through virtual glass panels displaying negative thoughts, symbolizing the act of overcoming doubts.

##### 2.2.1.8 Competition scene 4

This scene recreated the *end of the race* (the last 400 meters), where participants competed against *three* virtual runners. The virtual coach emphasized that this is the moment to strive for a personal best or top finish. While acknowledging the physical pain and exhaustion, the coach stressed that these would be temporary, but the memory of pushing limits and performing at one's best would last forever. The coach also highlighted that the smart pacing and drafting strategies used earlier would soon pay off. Upon crossing the finish line, participants were shown their position (ranging from first to fourth place).

##### 2.2.1.9 Coaching scene 5

In this scene, the virtual coach congratulated participants on completing the mental training session and encouraged them to apply the training in upcoming running competitions. The coach accentuated that mental training requires as much dedication as physical training. Finally, it provided instructions on how to exit, power down and recharge the VR headset, and reminded participants to fill out the study questionnaires. After leaving this scene, the setting scene reappeared.

#### 2.2.2 Log file

The application automatically generated or updated log files, recording scenes start and end times, participants' pacing, drafting scores, and VR training session timestamps. To prevent unauthorized modifications, the file was kept hidden and accessible only to members of the research team after the headsets were returned.

## 3 Methods

This section describes the recruited participants, procedure, materials, and open-ended data analysis.

### 3.1 Participants

This study included six participants (five men and one woman) from different nations, who were recreational regular runners of 5 km or longer races. Participants were between 26 and 35 years old and had an average of 5.5 years of running experience. Recruitment was voluntarily and based solely on participants' availability and willingness to complete the study, without restrictions regarding gender, age, education, or other criteria. No additional data were collected on professional background information or institutional affiliation.

All participants reported being aware of VR technology, though none were regular users. Recruitment was conducted through direct outreach and referrals (including contacts of the researchers and their acquaintances), rather than through institutional channels or online forums. Before enrollment, all participants were informed about the study's purpose and duration. The study was conducted in accordance with our research project's ethical declaration and was approved by the authors’ institutional ethics committee.

### 3.2 Procedure

This study, employing a single-subject A-B-A design (39), was structured into five consecutive phases: *first race phase, educational phase, on-boarding phase* (collectively forming the pre-intervention stage, A)*, VR training phase* (the intervention stage, B)*,* and *second race phase* (the post-intervention stage, A). The entire study spanned approximately *four weeks*, with scheduling adjusted to accommodate each participant's availability. As two participants lacked prior experience with smartwatches, they were provided with the devices (see Devices section) and received instructions on their operation to track their physical running performance.

#### 3.2.1 First race phase

This phase aimed to collect baseline data on participants' running performance prior to any mental VR training intervention. Each participant selected an official running race (first race or race 1) of their choice, ranging between 5 and 10 km. This range was chosen to compare training demands across participants, while also accommodating the practical constraint that identical race distances were rarely available within the study variable timeframe. Before the race, participants completed the Emotional Stress Reaction Questionnaire (ESRQ) (40). They then recorded their performance using a smartwatch, starting at the race's official start line and stopping at the finish. After finishing, participants completed the ESRQ again along with the Post-Race Questionnaire (PRQ) (See Self-assessment tools section). This phase therefore included the race itself and the completion of the questionnaires, all conducted on the same day.

#### 3.2.2 Educational phase

In this phase, the researcher provided only a concise introduction to the concepts of imagery, self-talk, and general running strategy, while deliberately excluding specific techniques like pacing and drafting. The aim was to give participants preliminary awareness rather than in-depth instruction or practice. The core intervention, where these concepts are contextualized and reinforced using VR, occurs in VR Training Phase. In the case of self-talk, participants received a collection of motivational self-talk phrases, to inspire their own creation of personalized self-talk statements for each race stage. These statements were designed to be concise, meaningful, and tailored to the specific demands of each stage. The entire phase lasted approximately one hour.

#### 3.2.3 On-boarding phase

During this phase, participants were instructed to independently use the VR device and its dedicated application for virtual training. The researcher provided a manual detailing the procedures for powering on, configuring, and shutting down the VR device, as well as accessing the VR application. Participants reviewed the manual and attempted to follow the instructions independently while the researcher remained available for assistance. Participants practiced the outlined steps repeatedly until they could operate both devices and applications without guidance. A copy of the manual was provided to each participant for future reference. The duration of this phase varied by individual, lasting between one and two hours.

#### 3.2.4 VR training phase

During this phase, participants independently trained race strategy (including pacing and drafting) and motivation using the VR application at home. They were required to complete a minimum of *nine* VR training sessions over three weeks. A *VR training session* was defined as the completion of all scene sequences within the VR application and lasted between 15 and 20 min, depending on the participant's interaction within the VR environment. For this phase, all participants received enough printed copies of the ESRQ, which they were instructed to complete before and after each session. Participants were asked to follow their regular physical training program alongside this VR training.

#### 3.2.5 Second race phase

This phase aimed to assess participants’ running performance after completing the VR training. Each participant selected a second official race (or race 2) between 5 and 10 km. As in the first race, participants first completed the ESRQ, then recorded their performance using a smartwatch from the official start line to the finish. After the race, they again completed the ESRQ along with the PRQ.

**Figure 3:**
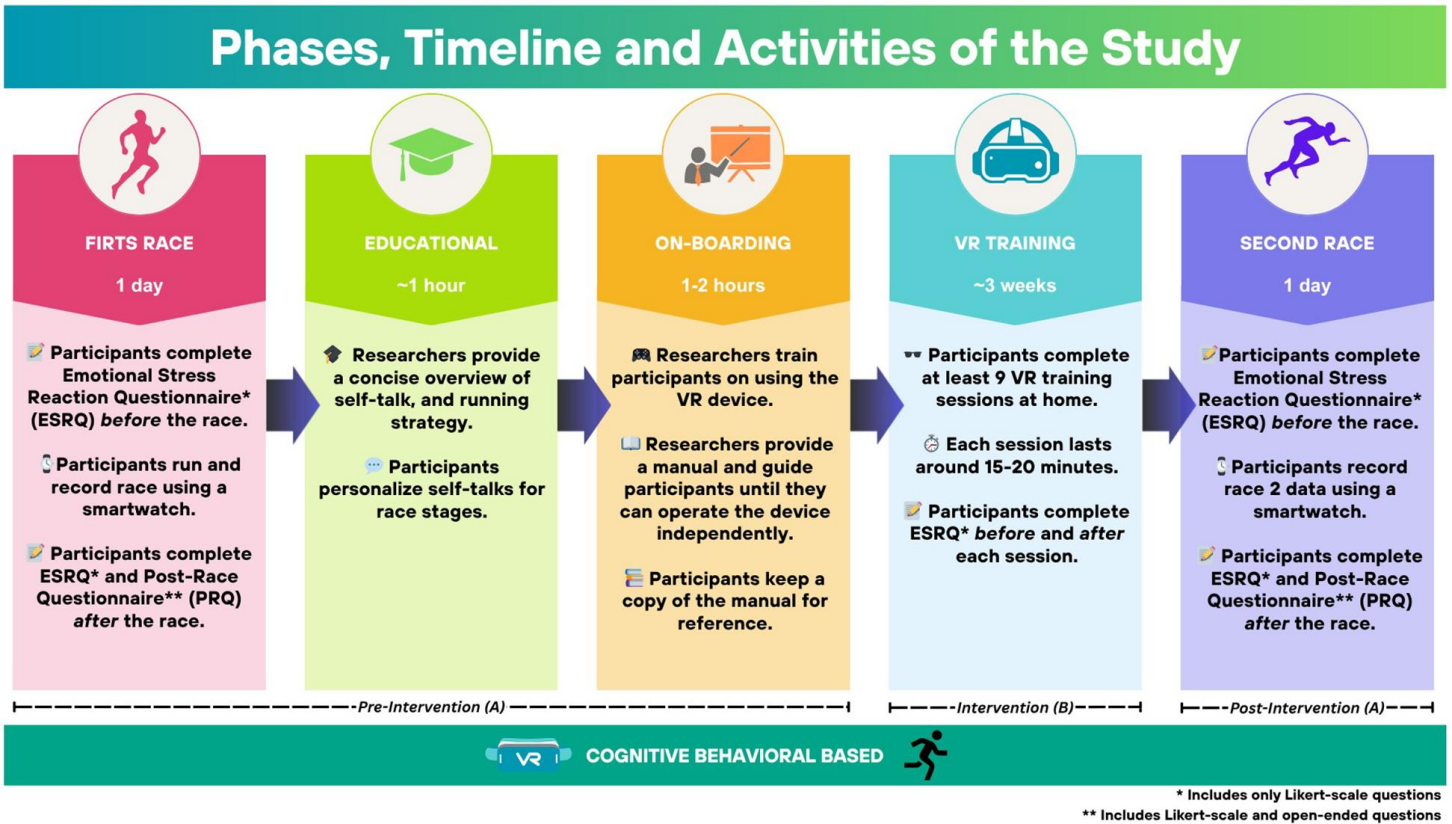
Phases, timeline and activities of the study.

Figure 3 provides an overview of the study phases, including their corresponding timeline and activities.

### 3.3 Materials

#### 3.3.1 Devices

Each participant received and used the *Meta Quest 2*, an all-in-one VR headset that offers full motion freedom and a high-resolution display. Participants who owned smartwatches used them to collect their running data, while those without smartwatches were provided with *TicWatch* devices.

The Strava application was chosen for its intuitive design, global use, and familiarity among some participants. It enabled the recording of parameters for evaluating participants' physical race performance, such as pacing and positions. After each race, participants stored this data and later shared it with the researchers.

#### 3.3.2 Self-assessment tools

Participants completed two self-assessment tools in English:

*Emotional Stress Reaction Questionnaire (ESRQ)* (40): This questionnaire comprises 14 questions on a 4-point Likert scale and was answered by participants before and after each VR training session, as well as before and after both actual races. Its purpose was to obtain the ESRQ-appraisal index, providing a snapshot of participants' emotions before and after their activities.
*Post-Race Questionnaire (PRQ)*: Adapted from the Post-Event Reflection Tool (41), this questionnaire was modified to align with the objectives of our study. It includes 32 open-ended questions, and seven items rated on a 10-point Likert scale (see Supplementary Material). The PQR assessed participants' evaluations of their performance and their impressions of key metrics throughout race stages (start, first half, second half, and end)*.* The metrics included *general impression, physical perception, motivation, self-talk, drafting,* and *pacing*.

### 3.4 Data analysis of open-ended responses

For the open-ended responses, we employed an inductive content analysis following a three-phase coding process (42, 43). First, raw open-ended responses were systematically *organized* according to the relevant metric and race stage. Next, *content analysis* was used to identify key descriptive words and phrases that reflected participants' meaning while preserving the integrity of their original statements. Finally, these descriptive elements were *synthesized* into concise coded categories that captured the essential qualitative insights in a clear and structured format.

## 4 Results

Table 1 presents the completed VR training sessions along with race metrics (distances, times and average speeds) for each participant (P). While P4 reported completing nine sessions, the VR device log files contained no recorded data, likely due to a malfunction. As a result, P4's VR session data is missing. However, P4 successfully completed all questionnaires and both races.

**Table 1:** VR training sessions & races metrics per participant.

*P*	Gender	VR training sessions	Race 1 (distance, time & average pacing)			Race 2 (distance, time & average pacing)		
			km	min:s	km/h	km	min:s	km/h
1	Male	9	5.4	27:16	11.9	4	24:56	9.5
2	Male	10	5	34:50	8.4	5	35:57	8.4
3	Male	9	10.2	53:32	11.5	10	53:47	11.2
4*	Male	9	9	55:15	9.8	4.9	28:29	10.2
5	Female	10	5.3	37:10	8.6	5	36:13	8.2
6	Male	10	9.5	41:38	13.7	5.4	22:57	14.1

* Missing VR session data for this participant.

### 4.1 VR training session performance: log file data

In the following two sections, we present the results of our analysis of the participants' log file data addressing *RQ1*.

#### 4.1.1 VR pacing

For each VR training session, the average pacing for all four competition scenes was calculated, converted to km/h, normalized, and plotted to identify characteristic pacing profiles.

**Figure 4:**
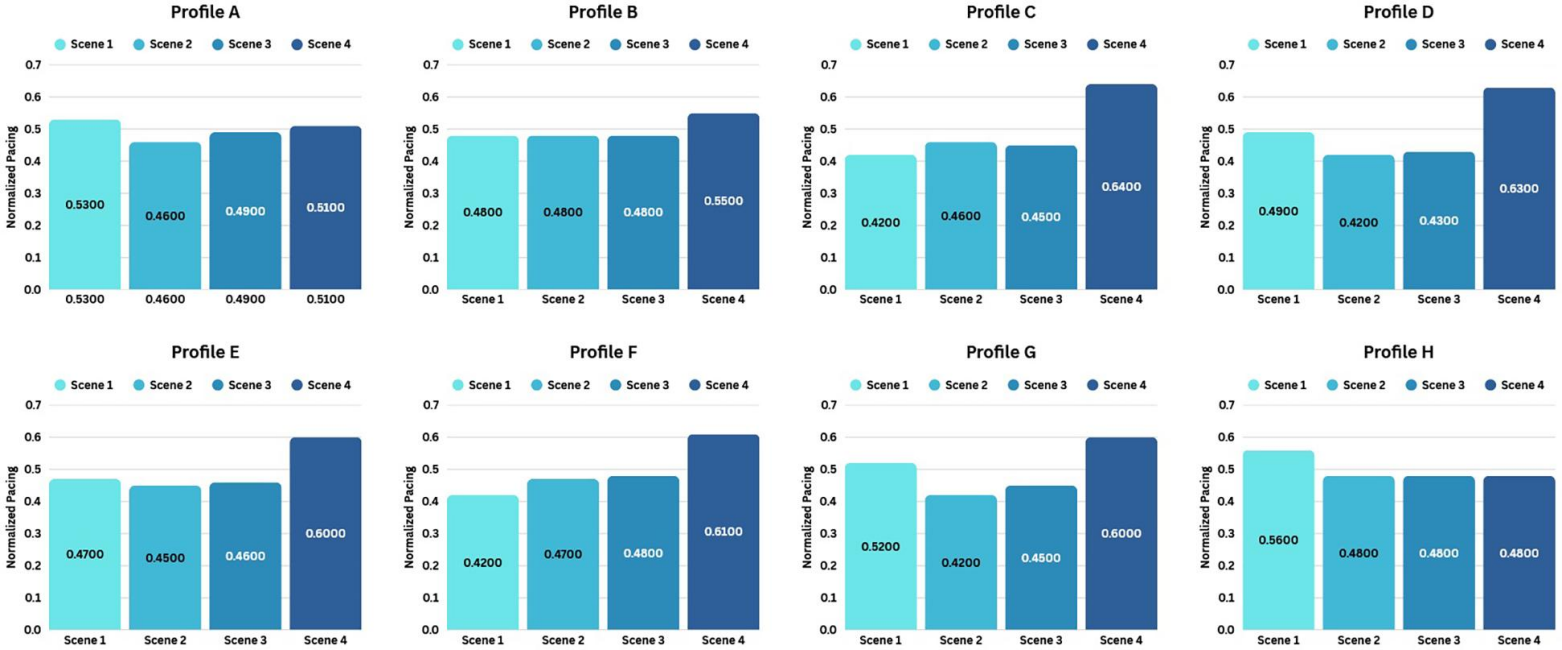
Most frequent normalized VR pacing profiles across Participants’ VR training sessions. The number within each bar indicates the normalized value of the scene's pacing.

Afterward, a total of 20 distinct pacing profiles were identified among all participants' VR training sessions (see Supplementary Material). Although each participant exhibited a unique set of profiles, certain patterns were more common, appearing in at least three different VR sessions across participants. The eight most frequently occurring normalized profiles are displayed in Figure 4. For instance, Profile D shows that the pacing in scene 1 was faster than in scenes 2 and 3 (with scene 3 faster than scene 2), but slower than in scene 4. In contrast, Profile F indicates a progressive increase in pacing across scenes, with scene 4 ultimately showing the fastest pacing.

#### 4.1.2 VR drafting

**Figure 5:**
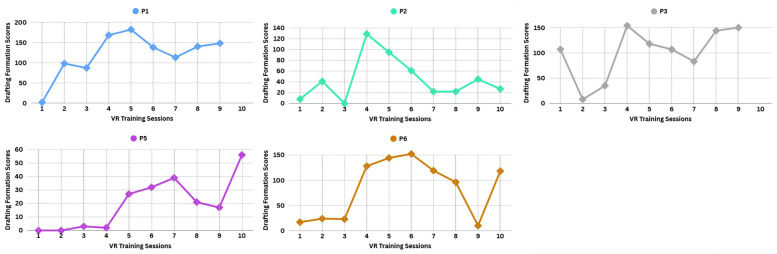
Participants’ drafting formation scores for each VR training session.

To evaluate VR drafting, we used the Drafting Formation Score (DFS). This score was calculated by summing the total points participants earned for positioning themselves in Formation-1 and Formation-2 (See Coaching Scene 2 section) during competition scenes 2 and 3. Participants had to maintain the correct formation for at least three seconds for the points to be counted. High DFS values indicate frequent engagement in drafting during VR training sessions. Figure 5 presents participants’ DFS results for each VR training session, revealing that most achieved their highest DFS values during sessions 4, 5, and 6 (peak performance). P1 obtained the highest overall DFS. P3 and P6 also exhibited relatively high engagement, with several high-scoring sessions. In contrast, P2 showed inconsistent performance, alternating between high and low DFS scores. P5 had the lowest overall engagement, with multiple near-zero scores, except for a few sessions of moderate improvement. Additionally, some participants, such as P2 and P6, had a decline in DFS after reaching their peak performance.

### 4.2 VR training session performance: ESRQ-appraisal Index data

**Figure 6:**
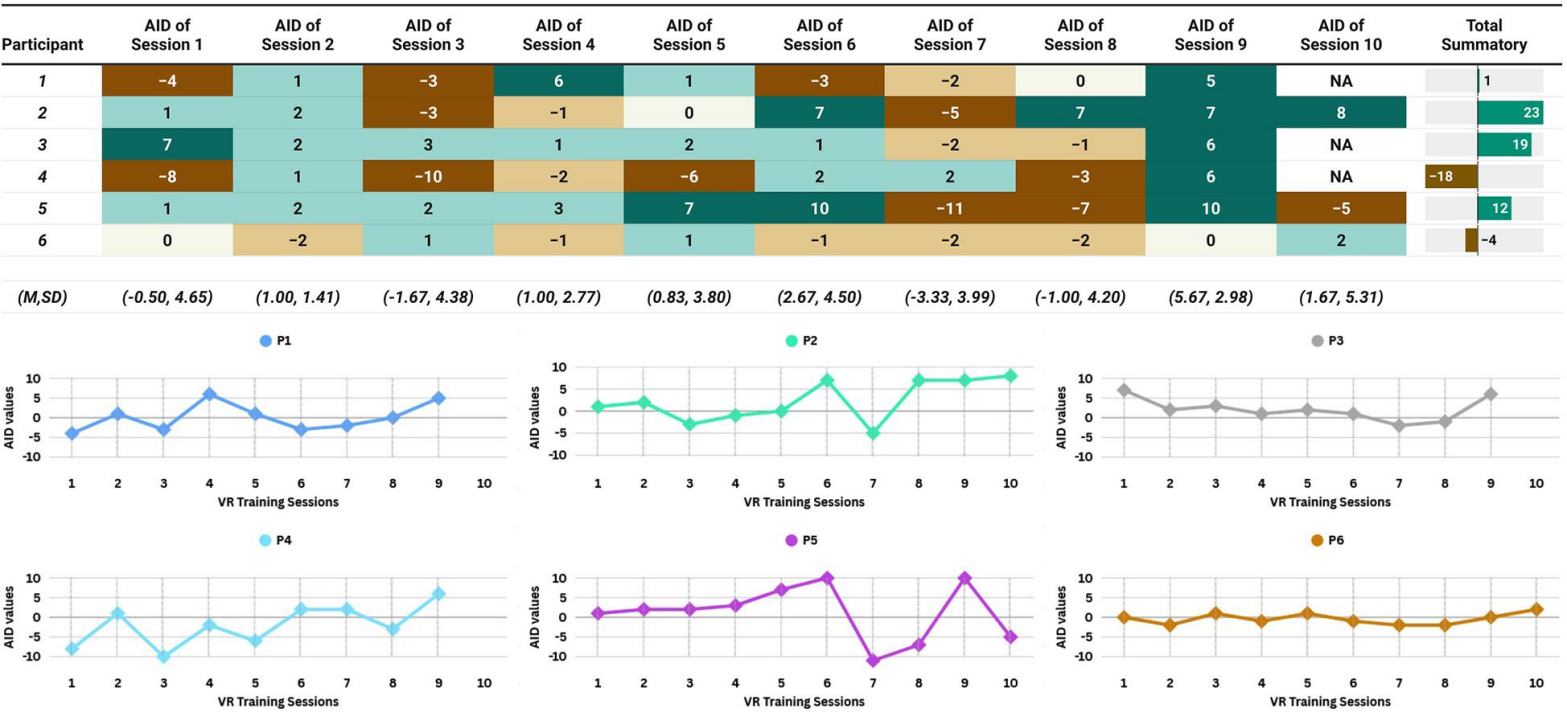
Participants’ appraisal Index difference (AID) across VR training sessions. (*Top*) Participants’ AID per VR Training Session and Their Corresponding Total Summatory. VR Training Session-wise Means (M) and Standard Deviations (SD). Color-Coding: Warmer greens = higher positive AID, deeper browns = lower negative AID (5-step color scale). (*Bottom*) Session-Based Visualization of the Participants’ AID Data Presented in the Top Panel.

To examine the influence of the VR application on participants' emotions (*RQ2*), we calculated the difference between the ESRQ-appraisal index after and before each VR training session. While the suitability of this metric for direct comparison is undefined, we computed it and referred to it as the ESRQ-Appraisal Index Difference (AID). The total summatory of all AID values for each participant was then obtained. Figure 6 shows participants' AID results for each VR training session. P2 and P3 exhibited the most consistent positive AID values, whereas P4 showed the most significant negative values. Overall, almost all participants had a positive or near-zero total AID summatory throughout their whole VR sessions.

### 4.3 Race performance: self-assessment data results

This section outlines participants' self-assessment responses for both races.

#### 4.3.1 ESRQ-appraisal index difference for races

**Figure 7:**
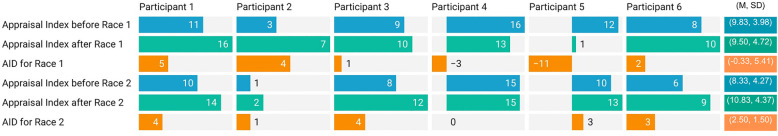
Participants’ appraisal Index difference (AID) for races. **Color-Coding**: Blue = pre-race appraisal index, Green = post-race appraisal index, Orange = AID for the race.

The AIDs for each participant's two races were calculated and are shown in Figure 7. This data was analyzed to partially address *RQ3*. In race 1, most participants had a positive AID, except for P4 and P5, who had negative values (−3 and −11, respectively). In race 2, most participants also experienced a positive AID, though P4 remained unchanged. Notably, P3 and P6 consistently had positive emotional responses after both races. P5, who had a low AID in race 1 (−11), improved significantly in race 2 (+3). On average, participants showed a higher AID in race 2 (M = 2.50, SD = 1.50) compared with race 1 (M = −0.33, SD = 5.41).

#### 4.3.2 PRQ-likert-scale questions

**Figure 8:**
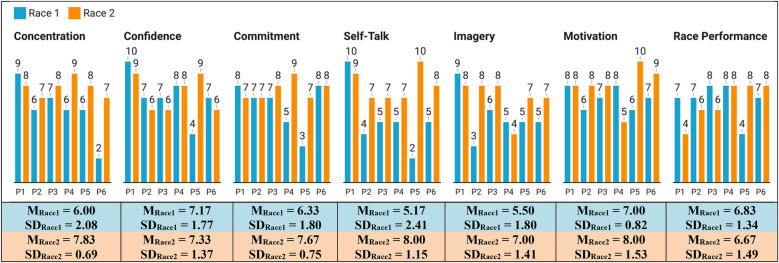
Results of the PRQ Likert-scale questions for races, including their means (M) and standard deviations (SD).

These questions provided insights into *concentration*, *confidence*, *commitment*, *self-talk*, *imagery*, *motivation* and *race performance*, thus partially addressing *RQ3*. The results of these metrics are presented in Figure 8. Based on these results, we highlight that P1 and P4 maintained high scores in multiple metrics in both races. P5 stood out with the most improvement across all metrics. Additionally, most participants showed improvement in metrics like *concentration*, *self-talk*, and *imagery* in race 2. However, a few participants experienced declines in specific metrics, such as P1 in *race performance*, and P4 in *motivation*.

#### 4.3.3 PRQ-open-ended questions

In this section, the following metrics are reported: *physical perception (RQ3), motivation (RQ3), self-talk (RQ3), pacing (RQ4),* and *drafting (RQ4)*.

##### 4.3.3.1 Physical perception

Regarding physical perception of the participants, we sorted their responses into three distinct groups: a) *positive physical perception*, b) *negative physical perception*, with the associated challenge or issue in parentheses, and c) *ambivalent physical perception*, with the associated challenge or issue in parentheses. Table 2 shows that most participants (except P1) felt good or fine at the start of both races. Aside from P1, participants reported feeling the same or better during the first half of race 2 compared to race 1. The second half and end of both races were the most physically demanding stages, with common challenges including *tiredness*, *leg discomfort* and *overheating*.

**Table 2:** Participants’ physical perception across race stages.

*P*	Race	Start	1st half	2nd half	End
1	1	Good	Good	Good	Great
	2	Bad (legs no reacting)	Fine (25% better)	Bad (tiredness)	Good
2	1	Good	Fine (overheated)	Fine (overheated & tired)	Bad (tiredness)
	2	Fine (leg pain)	Fine (leg pain)	Fine (tired & overheated)	Bad (high tiredness)
3	1	Fine (a bit overheated)	Good	Bad (overheated)	Fine (tired)
	2	Good	Good	Bad (overheated)	Good
4	1	Great	Great	Bad (increasing tiredness)	Bad (high tiredness)
	2	Fine (lacking sleep)	Good	Bad (high tiredness)	Bad (high tiredness)
5	1	Good	Fine (a bit tired)	Bad (abdominal pain)	Bad (tiredness & leg pain)
	2	Great	Great	Very bad (tiredness, nausea, rapid breathing)	Bad (less tiredness than before)
6	1	Fine (overheated)	Fine (tired & overheated)	Bad (tiredness & overheated)	Bad (tiredness and overheated)
	2	Good	Fine (high heart rate	Bad (heavy legs)	Bad (heavy legs & high heart rate)

**Color-Coding**: *Green* **=** Positive physical perception, *Blue* *=* Negative physical perception with the associated challenge or issue in parentheses, *Yellow* *=* Ambivalent physical perception with the associated challenge or issue in parentheses.

##### 4.3.3.2 Motivation

Table 3 presents the participants' responses on motivation across the races. We categorized these responses into three distinct groups: a) *positive motivation*, b) *neutral or strategic focus*, and c) *low motivation or challenges*. In the first half of both races, all participants reported sustained motivation, primarily by focusing on pacing, competition, or personal achievement. By the second half, however, nearly half experienced a decline, commonly attributed to fatigue, loss of drive, or a general sense of discouragement. P4 and P5 struggled to maintain motivation in the latter stages of both races. P3 and P4 reported intentionally applying VR strategies to enhance motivation; however, P4 continued to experience challenges in sustaining motivation despite using them. P1, P3, and P6 consistently maintained high motivation throughout both races, whereas others, such as P4 and P5, experienced difficulties.

**Table 3:** Participants’ motivation factors across race stages.

*P*	Race	Start	1st half	2nd half	End
1	1	Keep a controlled pacing	Keep a controlled pacing	Keep a controlled pacing	Keep training for next races
	2	Overcome the fatigue from previous competitions, set a good time	Keep a controlled pacing, avoid injuries	Set a good time	Finish the race
2	1	Competitive drive	Competitive drive	Finish the race	Finish the race
	2	Set a good time	Conserve energy	Keep a controlled pacing	Recover lost time
3	1	Have a good performance after harder races	Have a good performance after harder races	Competitive drive	Set a new personal best time
	2	Evaluate the VR strategies	Keep a controlled pacing	Keep a controlled pacing	Give all I had left
4	1	Keep a controlled pacing	Competitive drive	Loss of motivation	Low motivation
	2	Evaluate the VR strategies in the race	Achieve a good time by using VR strategies	Unmotivated because of tiredness	Finish the race
5	1	Competitive drive	Competitive drive	Unmotivated as many people overtook me	Finish the race
	2	Competitive drive	Competitive drive	Competitive drive but highly tired	Finish the race
6	1	Set a new personal best time	Set a new personal best time	Set a new personal best time	Set a new personal best time
	2	Set a good time after being sick	Keep the good start of the race	Finish the race	Keep the race position

**Color-Coding**: *Green* **=** Positive motivation, *Blue* *=* Neutral or strategic focus, *Yellow* *=* Low motivation or challenges.

By comparing the motivators mentioned by the participants at different stages of their races, we found that *pacing, time goals, competitive drive*, and *personal achievement* were strong motivators for most, particularly at the start and during the first half of the races.

##### 4.3.3.3 Self-talk

Table 4 presents the most significant features of self-talk utilized by participants across races. We classified them as instructional and motivational. All participants used self-talk during the second race, with most (five participants) incorporating similar versions or segments of the self-talk they heard during the VR training phase and applying them at the corresponding stage of the second race. In both races, self-talk in the second half mainly focused on overcoming fatigue or challenges (e.g., “You can do it”), while toward the end, it served as motivation for a strong finish (e.g., “Almost there”).

**Table 4:** Participants’ self-talk application across race stages.

*P*	Race	Start	1st half	2nd half	End
1	1	Run slowly	Keep it up	Keep it up	You did it
	2	Don't stop, you are a machine	Don't stop, you are a machine	Go to the end	You did it
2	1	Here it goes	No self-talk	Focus on breathing	Almost there
	2	Focus on breathing	Focus on breathing	Focus on breathing	Almost there
3	1	No self-talk	Keep pacing	Keep going through fatigue and heat	Keep pushing, you are in a good rhythm
	2	Keep calm, keep pacing	Run smart, keep it up	It will be over soon!	Finish strong
4	1	No self-talk	Overtake or lose	Almost done, keep the pace	Overtake this person! final stretch
	2	Take it easy	Take it easy, stay behind	You can do it!	Overtake this person!
5	1	I can do it	Less than when I started	Almost there! You can do it	Almost there!
	2	Slowly, on my rhythm	Slowly, on my rhythm	Less than when I started, let's go	Overtake the person in front!
6	1	Keep pacing and breathing	Slow down a bit	Jesus, help me	Get through it
	2	Keep calm, keep the pacing	Slow down a bit	Focus on yourself, keep a good technique	Almost there, give your best

**Color-Coding**: *Blue* = Instructional self-talk, *Green* = Motivational self-talk.

In race 1, participants used motivational self-talk more frequently than instructional self-talk (14 vs. 7 instances). By race 2, this pattern shifted, with instructional self-talk becoming more prevalent (14 instances vs. 10 instances). In both races, motivational self-talk remained common in later stages, particularly at the end, while instructional self-talk was more common in the early and middle stages to make decisions (“Overtake”) and regulate pacing or drafting (e.g., “Take it easy, stay behind”).

##### 4.3.3.4 Drafting

In race 1, P2, P3, P4, and P5 explicitly stated they had no understanding of drafting, while P1 and P6 had some knowledge but did not apply. After their VR training sessions, all participants reported using drafting in race 2 (Table 5). Drafting was most common at the start and the first half, with five out of six participants reported applying it at the start and all using it in the first half. However, its use declined in the second half, with only P1, P2, and P5 attempting but failing to sustain it. By the race' end, drafting significantly decreased, with only P2 and P5 maintaining it until the end. Overall, all participants reported attempting or applying drafting at some point in race 2, primarily in the start and first half. The most mentioned reason for failed drafting attempts was the absence of nearby runners.

**Table 5:** Participants’ Use of drafting across races stages.

*P*	Race	Start	1st half	2nd half	End
1	1	No drafting			
	2	Used	Used	Used	Attempted
2	1	No drafting			
	2	Used	Used	Attempted	Used
3	1	Not drafting			
	2	Used	Used	Attempted	No drafting
4	1	No drafting			
	2	Used	Used	No drafting	No drafting
5	1	No drafting			
	2	Used	Used	Attempted	Used
6	1	No drafting			
	2	No drafting	Used	Used	Attempted

##### 4.3.3.5 Pacing (self-reported and smartwatch data)

Table 6 presents participants’ self-reported pacing data (categorized as slow, controlled and fast) across race stages, alongside plots of their filter pacing data (using a moving average filter with a window of 15 distance units, km) obtained from the Strava application on their smartwatches. The self-reported pacing data partially addressed *RQ4* by assessing the possible influence of the VR training in the use of race strategy.

**Table 6:** Participants’ pacing across race stages.

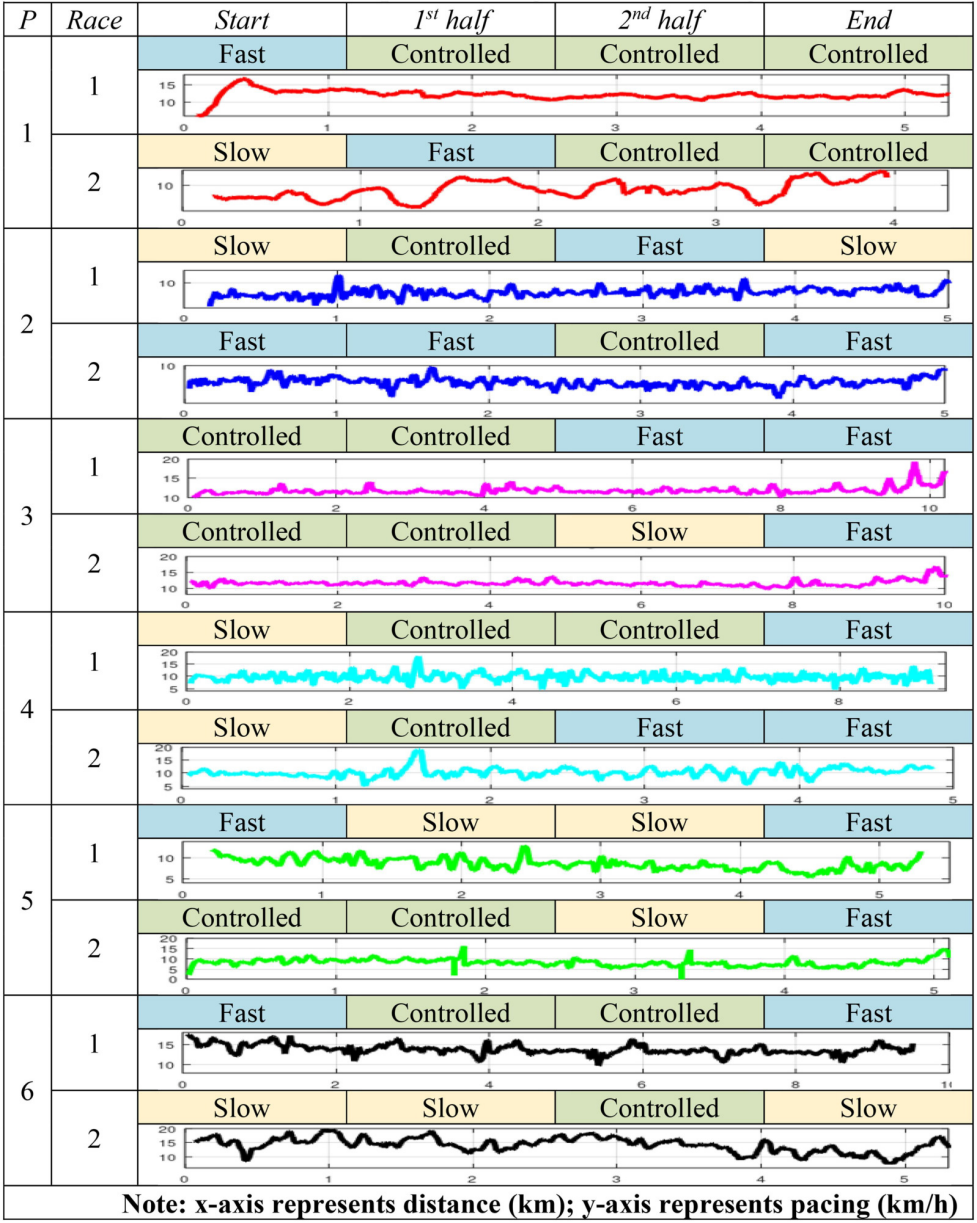

In race 1, all participants reported knowing the concept of pacing. Regarding the self-reported pacing data, most participants indicated maintaining a “controlled” pacing during segments of the race and finished strongly. P2, who reported starting race 1 with a “slow” pacing and finished similarly, mentioned adopting a more aggressive strategy in race 2 by starting “fast” and adjusting pacing strategically. P5, who reported inconsistent pacing in race 1, indicated having better control in race 2, starting with a “controlled” pacing and maintaining it through the first half. Unlike race 1, P3 and P6 reported a slowdown in the second half and toward the end of race 2.

Participants' smartwatch data showed variations in pacing across both races. In race 1, P1 started fast but faded, whereas in race 2, a more stable initial pacing was maintained. P2 transitioned from a steady pacing with a final sprint in race 1 to a more even pacing with a slight acceleration in race 2. P3 maintained a stable pacing in both races with a stronger final sprint in race 2. P4 showed consistent pacing across both races with minor adjustments. P5 slightly improved energy management in race 2, maintaining a more stable pacing at the start and first half. P6 showed a slightly better initial pacing in race 2 and finished stronger. Overall participants demonstrated improved pacing, or stronger finishes in race 2 compared to race 1. Those who struggled with early pacing in race 1 adjusted in race 2, while those who had already paced well maintained it.

When comparing self-reported data with its smartwatch counterpart, some discrepancies emerge due to fluctuations that categorical description fail to capture. For example, in race 2, P3 described the second half as “slow”, but the plot indicates a relatively stable pacing trend. Similarly, P6's race 2 plot shows greater variability than the “slow” labels suggest, particularly in the first half. In P2's case, while the “slow” label at the start aligns with the plot, the “fast” label in the second half does not match with the plot's variability.

## 5 Discussion

After employing a single-subject A-B-A design across recreational runners to evaluate the impact of our CB-based VR training, we address the research questions and cautiously discuss our exploratory findings.

Regarding **RQ1**, all participants (recreational runners) executed the instructed race strategy during VR training through a dynamic learning process, as reflected in trends in both VR pacing and VR drafting performance. In early sessions (1–3), variability in pacing profiles indicates experimentation with different pacing options, engaging in a trial-and-error process as they adapted to the VR environment and learned pacing control. By mid-to-late sessions (4–9/10), VR pacing becomes more consistent, suggesting that participants may have internalized a preferred, personalized pacing profile in the virtual environment, aligned with their cognitive responses.

For VR drafting, peak performance observed in sessions 4–6 (as reflected by high DFS values) suggests participants effectively applied drafting strategies during this period, likely due to heightened engagement and focused coaching-driven practice on formation adherence. Together, these trends in VR pacing and VR drafting may point to a plausible *four-session* learning benchmark for race strategy mastery within the VR training. However, declining scores in later sessions also imply challenges in sustaining this execution. Potential contributing factors include changes in motivation, shifts in attention (e.g., exploring the virtual environment), or cognitive overload from balancing pacing and drafting, factors that have been noted in previous literature (44–46). Therefore, this highlights that while participants may initially master VR drafting, preserving this skill with precision over time could require a more adaptive virtual training design, rooted in coaching methodologies, that could dynamically adjust as DFS values decline in order to sustain participants' engagement.

For **RQ2**, regarding the impact of the CB-based VR application on participants' self-reported emotional reactivity during the VR training phase, our exploratory results indicated a moderate positive effect, though individual responses varied. In many cases, self-reported emotional states showed improvement over sessions, possibly reflecting some degree of adaptation or increased confidence with repeated VR exposure. This pattern, however, was not consistent across all participants (recreational runners); in two cases, emotional reactivity changes appeared minimal or negligible. Such variability may relate to individual factors affecting responsiveness to VR training, including the timing of VR training sessions or differences in fatigue levels (47, 48).

Regarding **RQ3**, VR training sessions appeared to relate to changes in participants' self-assessments of psychological factors, as reflected by shifts in emotional reactions, CB skills, mental toughness attributes and race perceptions. For CB skills, participants reported perceived gains in imagery and, particularly, self-talk during race 2, as shown in PRQ Likert-scale responses. All participants noted some use of self-talk in their second race, with a greater reported frequency of instructional self-talk compared to race 1, a finding complemented by open-ended responses. These observations may indicate that the CB-based VR training may have supported an increased use of self-talk in the second race, although the extent of its influence remains uncertain. Prior VR research has shown that immersive, repeated practice environments can foster the adoption of self-talk strategies and enhance their integration into performance contexts (48, 49).

Mental toughness attributes, such as confidence and commitment, showed mixed patterns highlighting possible areas for more targeted coaching in future program designs. Perceptions of motivation revealed two general trends: most participants began race 2 with heightened strategic focus or positive motivation, this often declined in later stages due to physical fatigue, a pattern also observed in race 1. PRQ open-ended responses identified tiredness as a primary factor in this decline, aligning with physical perception metric.

For **RQ4**, based on the reported data, VR training appeared to influence participants' self-reported use of running strategy in the second race. For pacing, self-reported data from PRQ-open-ended questions indicated that most participants, who were recreational runners, perceived themselves as maintaining a “controlled” pacing across both halves of the races with a “fast” finish. However, smartwatch metrics suggested that some participants exhibited slightly more consistent pacing in race 2, with fewer abrupt speed fluctuations compared to race 1.

Self-reported use of drafting strategies showed a more noticeable shift. While drafting was largely unknown or unused in race 1, by race 2 participants reported applying drafting technique in at least two race stages, including at the start and finish, despite these phases not being explicitly emphasized in the VR training phase. This may indicate that VR training played a role in the adoption of the drafting technique. Considering the limited scope and single-session format of the educational phase, it seems plausible that the structured, multi-session VR training phase contributed substantially, nevertheless the relative influence of each phase cannot be determined with certainty. For the parts where participants applied drafting in race 2 that were not explicitly trained in the VR training phase, this may reflect their ability to generalize principles learned in VR or adopt strategies based on perceived performance benefits during competition. This aligns with previous VR research showing that athletes can transfer learned skills to real-world contexts, even in scenarios not directly trained in the virtual environment (16).

This study acknowledges several limitations that constrain the generalizability of its findings and the ability to isolate specific effects, which are to be expected in single-subject design studies (50). The small participant cohort, consisting of only recreational runners and further reduced by missing data for one individual and an unbalanced gender distribution, restricts the breadth of general conclusions that can be drawn. Race performance is also inherently multifactorial, influenced not only by mental aspects but also by physical, technical, and body awareness factors (1), as well as nutrition, weather, terrain, and injury status (51–54). Methodological constraints, including variability in training schedules, the suitability of the self-assessment tools and the participant recruitment employed, and differences in race distances for some participants, add further complexity. Additionally, the audio component, particularly background music and the coach's emphasis on mental training in the final scene, may have influenced participants' motivation scores. Consequently, it is challenging to precisely attribute performance outcomes solely to the CB-based VR mental training, as its contribution is likely entangled with other influences.

Nevertheless, given the exploratory nature of our study and its use of a single-subject design, an approach that has supported the development and evaluation of unique interventions in sport psychology through repeated measurements over time (55), the study provided interesting insights into individual responses and highlighted the potential of CB-based VR training rather than aiming to make broad statistical claims.

## 6 Conclusions and future work

Our exploratory findings with a sample of recreational runners hint at the potential of CB-based VR training as a complementary component to traditional coaching programs, particularly for developing mental skills, an area often underemphasized in race preparation.

Results from our study suggest that integrating VR training with a CB-based application may offer a practical bridge between virtual preparation and real-world execution in sports coaching, particularly in running. For some participants, the intervention appeared to help apply certain strategies from the VR training sessions (such as instructional self-talk for pacing and drafting in early race stages, and motivational self-talk for managing fatigue toward the end) during actual competition. These strategies may have supported better energy management and focus for some individuals. While promising, this potential contribution requires further verification through future research.

The use of the CB-based VR application appeared to have a neutral to moderately positive influence for most participants, with self-reported emotional equilibrium maintained or slightly improved across sessions. The observed variability between individuals suggests that tailoring virtual coaching to individual psychological profiles could help optimize any potential benefits of VR-based approaches, though this remains unexplored.

In this exploratory investigation, a tentative pattern emerged in which four VR sessions appeared sufficient for participants to reach peak drafting proficiency and stabilize pacing patterns within the virtual environment. Declines in drafting performance after this point may indicate a need for individualized coaching adjustments to sustain progress, pointing to VR's potential as a flexible coaching tool that could benefit from adaptive program design.

Overall, these findings should be interpreted as exploratory and iterative and must be considered cautiously. They provide a preliminary foundation for refining VR training tools, with future research needed to disentangle VR's specific effects from other training variables, validate the tentative “four-session benchmark” observed here, and investigate adaptive protocols for individualized skill development. Future studies could also explore VR simulations that replicate late-race fatigue, adverse weather conditions by incorporating technologies such as haptic vests, gloves, or thermoregulation systems. Virtual scenarios could also be dynamically adapted to athletes' performance and include unforeseen challenges, such faster opponents, poor self-assessment of pacing or early-race injury, to better train coping strategies under pressure. Additionally, future research should incorporate usability metrics, such as the Assessing Determinants of Prospective Uptake of Virtual Reality Instrument (56), during data collection to better evaluate the multiple factors influencing the adoption of CB-based VR interventions.

As this research progresses, we propose developing an adaptive tutorial system within the VR application, allowing experienced users to skip repetitive instructions in subsequent sessions. To implement this feature, detailed analysis of performance metrics (especially those related to drafting or pacing) across VR training sessions would help determine how well users learn and retain race strategies. These insights could validate the efficiency of the training protocol and offer empirical guidance on optimizing the number of required sessions, ensuring the study design remains both effective and time-efficient.

Addressing these facets might enhance CB-based VR training by fostering individual adaptation and supporting holistic coaching programs that integrate both mental and physical aspects of athlete development. In this way, CB-based VR training can complement the expertise of coaches and sport psychologists by providing reproducible training experiences and improving accessibility for personalized athlete development. However, it should be viewed as an adjunctive approach that enriches, rather than replaces, traditional psychological or coaching practices.

Until further evidence is available, CB-based VR training should be viewed as *one component* of a potential holistic coaching approach, not a standalone solution.

## Data Availability

The original contributions presented in the study are included in the article/Supplementary Material, further inquiries can be directed to the corresponding authors.

## References

[1] Cardenas Hernandez FP Schneider J Di Mitri D Jivet I Drachsler H. Beyond hard workout: a multimodal framework for personalised running training with immersive technologies. Br J Educ Technol. (2024) 55:1528–59. 10.1111/bjet.13445

[2] Michalski SC Szpak A Saredakis D Ross TJ Billinghurst M Loetscher T. Getting your game on: using virtual reality to improve real table tennis skills. PLoS One. (2019) 14(9):e0222351. 10.1371/journal.pone.0222351 31504070 PMC6736297

[3] Slater M. Immersion and the illusion of presence in virtual reality. Br J Psychol. (2018) 109(3):431–3. 10.1111/bjop.12305 29781508

[4] Riva G Wiederhold BK Mantovani F. Neuroscience of virtual reality: from virtual exposure to embodied medicine. Cyberpsychol Behav Soc Netw. (2019) 22(1):82–96. 10.1089/cyber.2017.29099.gri 30183347 PMC6354552

[5] Egiziano M Chomienne L Hervet V Mascret N Kulpa R Montagne G. Virtual reality as a perceptual-motor training tool: validity and fidelity assessments of a 4×100 m relay simulator. Appl Sci. (2025) 15(6):3224. 10.3390/app15063224

[6] Montagne G Mascret N Bossard M Chomienne L Ledouit S Rao G An interdisciplinary framework to optimize the anticipation skills of high-level athletes using virtual reality. Front Sports Act Living. (2024) 6:1324016. 10.3389/fspor.2024.1324016 38410354 PMC10895038

[7] Wood G Wright DJ Harris D Pal A Franklin ZC Vine SJ. Testing the construct validity of a soccer-specific virtual reality simulator using novice, academy, and professional soccer players. Virtual Real. (2021) 25(1):43–51. 10.1007/s10055-020-00441-x

[8] Kittel A Lindsay R Le Noury P Wilkins L. The use of extended reality technologies in sport perceptual-cognitive skill research: a systematic scoping review. Sports Med Open. (2024) 10(1):128. 10.1186/s40798-024-00794-6 39612099 PMC11607248

[9] Tabbaa L Ang CS Siriaraya P She WJ Prigerson HG. A reflection on virtual reality design for psychological, cognitive and behavioral interventions: design needs, opportunities and challenges. Int J Hum Comput Interact. (2021) 37(9):851–66. 10.1080/10447318.2020.1848161

[10] Beck JS. Cognitive Behavior Therapy: Basics and Beyond. 2nd ed. New York: Guilford Press (2011).

[11] Lindner P Hamilton W Miloff A Carlbring P. How to treat depression with low-intensity virtual reality interventions: perspectives on translating cognitive behavioral techniques into the virtual reality modality and how to make anti-depressive use of virtual reality–unique experiences. Front Psychiatry. (2019) 10:792. 10.3389/fpsyt.2019.00792 31736809 PMC6836923

[12] Jennings AN Soder HE Wardle MC Schmitz JM Vujanovic AA. Objective analysis of language use in cognitive-behavioral therapy: associations with symptom change in adults with co-occurring substance use disorders and posttraumatic stress. Cogn Behav Ther. (2021) 50(2):89–103. 10.1080/16506073.2020.1819865 33021143 PMC7897212

[13] Gustafsson H Lundqvist C Tod D. Cognitive behavioral intervention in sport psychology: a case illustration of the exposure method with an elite athlete. J Sport Psychol Action. (2017) 8(3):152–62. 10.1080/21520704.2016.1235649

[14] Martin KA Moritz SE Hall CR. Imagery use in sport: a literature review and applied model. Sport Psychol. (1999) 13(3):245–68. 10.1123/tsp.13.3.245

[15] McCormick A Hatzigeorgiadis A. Self-talk and endurance performance. In: Meijen C, editor. Endurance Performance in Sport: Psychological Theory and Interventions. London: Routledge (2019). p. 153–67.

[16] Richlan F Weiß M Kastner P Braid J. Virtual training, real effects: a narrative review on sports performance enhancement through interventions in virtual reality. Front Psychol. (2023) 14:1240790. 10.3389/fpsyg.2023.1240790 37928573 PMC10622803

[17] Mouatt B Smith AE Mellow ML Parfitt G Smith RT Stanton TR. The use of virtual reality to influence motivation, affect, enjoyment, and engagement during exercise: a scoping review. Front Virtual Real. (2020) 1:564664. 10.3389/frvir.2020.564664

[18] Vallerand RJ. Intrinsic and extrinsic motivation in sport and physical activity: a review and a Look at the future. In: Tenenbaum G Eklund RC, editors. Handbook of Sport Psychology. Hoboken: John Wiley & Sons, Inc. (2012). p. 59–83. 10.1002/9781118270011.ch3

[19] Almagro BJ Sáenz-López P Fierro-Suero S Conde C. Perceived performance, intrinsic motivation and adherence in athletes. Int J Environ Res Public Health. (2020) 17(24):9441. 10.3390/ijerph17249441 33339278 PMC7767293

[20] Barnicle SP Burton D. Enhancing collegiate women’s soccer psychosocial and performance outcomes by promoting intrinsic sources of sport enjoyment. J Sports Sci Med. (2016) 15(4):678–87. 27928214 PMC5131222

[21] Gréhaigne J-F Godbout P Bouthier D. The foundations of tactics and strategy in team sports. J Teach Phys Educ. (1999) 18(2):159–74. 10.1123/jtpe.18.2.159

[22] Abbiss CR Laursen PB. Describing and understanding pacing strategies during athletic competition. Sports Med. (2008) 38(3):239–52. 10.2165/00007256-200838030-00004 18278984

[23] Beaumont F Bogard F Murer S Polidori G Madaci F Taiar R. How does aerodynamics influence physiological responses in middle-distance running drafting? Math Model Eng Prob. (2019) 6(1):129–35. 10.18280/mmep.060117

[24] Lundin RM Yeap Y Menkes DB. Adverse effects of virtual and augmented reality interventions in psychiatry: systematic review. JMIR Ment Health. (2023) 10:e43240. 10.2196/43240 37145841 PMC10199391

[25] Vatsal R Mishra S Thareja R Chakrabarty M Sharma O Shukla J. An analysis of physiological and psychological responses in virtual reality and flat screen gaming. IEEE Trans Affect Comput. (2024) 15(3):1696–710. 10.1109/TAFFC.2024.3368703

[26] Paivio A. Cognitive and motivational functions of imagery in human performance. Can J Appl Sport Sci. (1985) 10(4):22S–8. 4085129

[27] Polaha J Allen K Studley B. Self-Monitoring as an intervention to decrease swimmers’ stroke counts. Behav Modif. (2004) 28(2):261–75. 10.1177/0145445503259280 14997952

[28] Lerner BS Ostrow AC Yura MT Etzel EF. The effects of goal-setting and imagery training programs on the free-throw performance of female collegiate basketball players. Sport Psychol. (1996) 10(4):382–97. 10.1123/tsp.10.4.382

[29] Pates J Maynar I Westbury T. An investigation into the effects of hypnosis on basketball performance. J Appl Sport Psychol. (2001) 13(1):84–102. 10.1080/10413200109339005

[30] Xu N Yan W Sun M Li R Wang Y Chi L. Applying skill-oriented and spirit-oriented psychological training to Chinese national archery team athletes: a single-case mixed-methods study. Int J Sport Exerc Psychol. (2024) 22(4):926–52. 10.1080/1612197X.2023.2168724

[31] Loehr JE. Mental Toughness Training for Sports: Achieving Athletic Excellence. Lexington: S. Greene Press (1986).

[32] Botella C Fernández-Álvarez J Guillén V García-Palacios A Baños R. Recent progress in virtual reality exposure therapy for phobias: a systematic review. Curr Psychiatry Rep. (2017) 19(7):42. 10.1007/s11920-017-0788-4 28540594

[33] Freitas JRS Velosa VHS Abreu LTN Jardim RL Santos JAV Peres B Virtual reality exposure treatment in phobias: a systematic review. Psychiatric Q. (2021) 92(4):1685–710. 10.1007/s11126-021-09935-6 34173160

[34] Opriş D Pintea S García-Palacios A Botella C Szamosközi Ş David D. Virtual reality exposure therapy in anxiety disorders: a quantitative meta-analysis. Depress Anxiety. (2012) 29(2):85–93. 10.1002/da.20910 22065564

[35] Turman ML Olden M Emrich M Difede J. Virtual Reality Exposure Therapy. Cham: Springer (2024). p. 63–84. 10.1007/978-3-031-72720-7_4

[36] van Loenen I Scholten W Muntingh A Smit J Batelaan N. The effectiveness of virtual reality exposure–based cognitive behavioral therapy for severe anxiety disorders, obsessive-compulsive disorder, and posttraumatic stress disorder: meta-analysis. J Med Internet Res. (2022) 24(2):e26736. 10.2196/26736 35142632 PMC8874794

[37] Stanney K Lawson BD Rokers B Dennison M Fidopiastis C Stoffregen T Identifying causes of and solutions for cybersickness in immersive technology: reformulation of a research and development agenda. Int J Hum Comput Interact. (2020) 36(19):1783–803. 10.1080/10447318.2020.1828535

[38] Cardenas Hernandez F Schneider J Di Mitri D Drachsler H. On-Your marks, ready? Exploring the user experience of a VR application for runners with cognitive-behavioral influences. Proceedings of the 17th International Conference on Computer Supported Education (2025). p. 331–41. 10.5220/0013271300003932

[39] Engel RJ Schutt RK. Fundamentals of Social Work Research. Thousand Oaks: Sage Publications (2010).

[40] Larsson G. The emotional stress reaction questionnaire (ESRQ): measurement of stress reaction level in field conditions in 60 s’. Proceedings of NATO HFM-205 Mental Health and Well-Being Across the Military Spectrum (2011).

[41] Chow GM Luzzeri M. Post-event reflection: a tool to facilitate self-awareness, self-monitoring, and self-regulation in athletes. J Sport Psychol Action. (2019) 10(2):106–18. 10.1080/21520704.2018.1555565

[42] Elo S Kyngäs H. The qualitative content analysis process. J Adv Nurs. (2008) 62(1):107–15. 10.1111/j.1365-2648.2007.04569.x 18352969

[43] Miles MB Huberman AM. Qualitative Data Analysis: An Expanded Sourcebook. 2nd ed. Thousand Oaks: SAGE Publications (1994).

[44] Han Y Diao Y Yin Z Jin R Kangwa J Ebohon OJ. Immersive technology-driven investigations on influence factors of cognitive load incurred in construction site hazard recognition, analysis and decision making. Adv Eng Inform. (2021) 48:101298. 10.1016/j.aei.2021.101298

[45] Huang W Roscoe RD Johnson-Glenberg MC Craig SD. Motivation, engagement, and performance across multiple virtual reality sessions and levels of immersion. J Comput Assist Learn. (2021) 37(3):745–58. 10.1111/jcal.12520

[46] Voinescu A Fodor LA Fraser DS David D. Exploring Attention in VR: Effects of Visual and Auditory Modalities. Cham: Springer (2020). p. 677–83. 10.1007/978-3-030-51828-8_89

[47] Cumpanas AA Bardan R Ferician O Latcu SC Lazar OF Duta C. The impact of tiredness on virtual reality robotic surgical skills. Videosurg Other Miniinvasive Tech. (2020) 15:298–304. 10.5114/wiitm.2020.93201 32489490 PMC7233167

[48] Ratan R Lin Q Lim C Park R Lover A Han E Time matters in VR: students benefit from longer VR class duration, but certain outcomes decline after 45 min, with large individual variance. Comput Educ. (2025) 235:105328. 10.1016/j.compedu.2025.105328

[49] Slater M Neyret S Johnston T Iruretagoyena G Crespo MÁC Alabèrnia-Segura M An experimental study of a virtual reality counselling paradigm using embodied self-dialogue. Sci Rep. (2019) 9(1):10903. 10.1038/s41598-019-46877-3 31358846 PMC6662659

[50] Walker SG Carr JE. Generality of findings from single-case designs: it’s not all about the “N.”. Behav Anal Pract. (2021) 14(4):991–5. 10.1007/s40617-020-00547-3 34868812 PMC8586328

[51] Jiménez-Alfageme R Garrone FP Rodriguez-Sanchez N Romero-García D Sospedra I Giménez-Monzó D Nutritional intake and timing of marathon runners: influence of athlete’s characteristics and fueling practices on finishing time. Sports Med Open. (2025) 11(1):26. 10.1186/s40798-024-00801-w 40089940 PMC11911277

[52] Lemire M Falbriard M Aminian K Millet GP Meyer F. Level, uphill, and downhill running economy values are correlated except on steep slopes. Front Physiol. (2021) 12:697315. 10.3389/fphys.2021.697315 34276417 PMC8281813

[53] Mantzios K Ioannou LG Panagiotaki Z Ziaka S Périard JD Racinais S Effects of weather parameters on endurance running performance: discipline-specific analysis of 1,258 races. Med Sci Sports Exerc. (2022) 54(1):153–61. 10.1249/MSS.0000000000002769 34652333 PMC8677617

[54] McGrath TM Fontana MA Toresdahl BG. Injury patterns and healthcare utilisation by runners of the New York city marathon. BMJ Open Sport Exerc Med. (2024) 10(1):e001766. 10.1136/bmjsem-2023-001766 38562153 PMC10982772

[55] Barker JB Mellalieu SD McCarthy PJ Jones MV Moran A. A review of single-case research in sport psychology 1997–2012: research trends and future directions. J Appl Sport Psychol. (2013) 25(1):4–32. 10.1080/10413200.2012.709579

[56] Glegg SMN Holsti L Stanton S Hanna S Velikonja D Ansley B Evaluating change in virtual reality adoption for brain injury rehabilitation following knowledge translation. Disabil Rehabil Assist Technol. (2017) 12(3):217–26. 10.3109/17483107.2015.1111944 28508725

[57] Neumann DL Moffitt RL Thomas PR Loveday K Watling DP Lombard CL A systematic review of the application of interactive virtual reality to sport. Virtual Real. (2018) 22(3):183–98. 10.1007/s10055-017-0320-5

[58] Yunchao M Mengyao R Xingman L. Application of virtual simulation technology in sports decision training: a systematic review. Front Psychol. (2023) 14:1164117. 10.3389/fpsyg.2023.1164117 37275736 PMC10232800

