# Supplementary material for: Miles with mind: an exploratory study of cognitive-behavioral-based VR training for strategy and motivation in long-distance running
Source: Front Sports Act Living. 2025 Nov 28;7:1722120. doi: 10.3389/fspor.2025.1722120 (PMC12698523; doi:10.3389/fspor.2025.1722120)
Supplement: Supplementary file 1 [file Datasheet1.pdf]

Supplementary Material

1 Supplementary Data

1.1 The Post-Race Questionnaire (PRQ)

Adapted from (Chow & Luzzeri, 2019)

Event: __________ km running race

Date: ________________________________ Time: _______________________________

**1. How would you rate your running performance today?**										
0 ○	1 ○	2 ○	3 ○	4 ○	5 ○	6 ○	7 ○	8 ○	9○	10 ○
Poor					Acceptable					Excellent

2. For each concept, please recount your experiences in relation to the specific phase of the running race you were in at that time (if you are unsure of the answer, please leave the space blank).

Start of the race (from 0 to 1/5 of the total distance)

What was your general impression?

____

____

What can you say about your motivation? Please, explain in detail

____

____

Do you remember speaking to yourself internally (Self-talk)?
If yes, what phrases did you use?

____

____

How did you feel physically?

____

____

Can you say anything about your pacing?

____

____

Can you say anything about drafting?

____

Did you play any song or melody in your head? If yes, which one was it?

1st half of the race (from 1/5 to 2/5 of the total distance)

What was your general impression?

What can you say about your motivation? Please, explain in detail

Do you remember speaking to yourself internally (Self-talk)?
If yes, what phrases did you use?

How did you feel physically?

Can you say anything about your pacing?

Can you say anything about drafting?

Did you play any song or melody in your head? If yes, which one was it?

2nd half of the race (from 2/5 to 4/5 of the total distance)

What was your general impression?

____

____

What can you say about your motivation? Please, explain in detail

____

____

Do you remember speaking to yourself internally (Self-talk)?
If yes, what phrases did you use?

____

____

How did you feel physically?

____

____

Can you say anything about your pacing?

____

____

Can you say anything about drafting?

____

____

Did you play any song or melody in your head? If yes, which one was it?

____

____

End of the race (last 400 meters)

What was your general impression?

____

____

What can you say about your motivation? Please, explain in detail

____

____

Do you remember speaking to yourself internally (Self-talk)?
If yes, what phrases did you use?

____

____

How did you feel physically?

___

___

Can you say anything about your pacing?

___

___

Can you say anything about drafting?

___

___

Did you play any song or melody in your head? If yes, which one was it?

___

___

3. Please provide an answer to the following questions, elaborating further whenever possible

a) *Did your goals change during the race? If so, how? (For example, I planned to finish in a high position, or simply complete the race)*

___

___

b) *During the race, were you able to identify or recognize any of the visual or auditory cues that were part of the VR training scenarios? If so, which ones?*

___

___

c) *Did you engage in self-talk during the actual race, and if so, how did it manifest?*

___

___

4. Rate your mental attributes and skills after running today			
	**Low**	**OK**	**High**
Concentration	0○ 1○ 2○ 3○ 4○	5○ 6○ 7○	8○ 9○ 10○
Confidence	0○ 1○ 2○ 3○ 4○	5○ 6○ 7○	8○ 9○ 10○
Commitment	0○ 1○ 2○ 3○ 4○	5○ 6○ 7○	8○ 9○ 10○
Self-Talk	0○ 1○ 2○ 3○ 4○	5○ 6○ 7○	8○ 9○ 10○
Imagery	0○ 1○ 2○ 3○ 4○	5○ 6○ 7○	8○ 9○ 10○
Motivation	0○ 1○ 2○ 3○ 4○	5○ 6○ 7○	8○ 9○ 10○

5. Anything else you want to note?

____

____

2 Supplementary Figures and Tables

2.1 Supplementary Figures

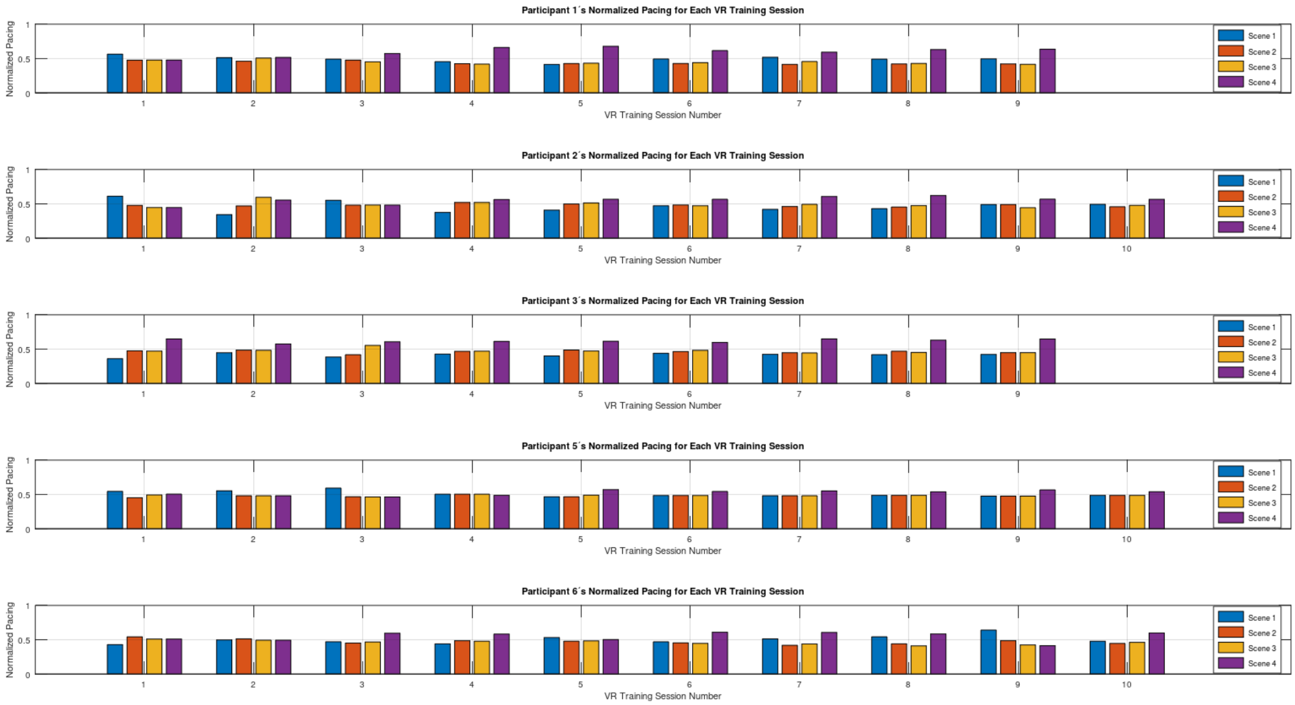

Supplementary Figure 1. Normalized VR pacing profiles of participants during their VR training sessions.
